# Cold snaring vs. standard forceps biopsy in sampling colorectal cancer: a comparative case report

**DOI:** 10.1055/a-2535-1929

**Published:** 2025-02-20

**Authors:** Simona Agazzi, Eukene Rojo, Elena De Cristofaro, Jérôme Rivory, Thomas Walter, Tanguy Fenouil, Mathieu Pioche

**Affiliations:** 1 18631 Gastroenterology and Digestive Endoscopy Unit, Fondazione IRCCS Policlinico San Matteo, Pavia, Italy; 2 16517 Gastroenterology Unit, Hospital Universitario de la Princesa, Madrid, Spain; 3 9318 Gastroenterology Unit, Department of Systems Medicine, University of Rome Tor Vergata, Rome, Italy; 4 Gastroenterology and Endoscopy Unit, Edouard Herriot Hospital, Hospices Civils de Lyon, Lyon, France; 5 Digestive Oncology Unit, Edouard Herriot Hospital, Hospices Civils de Lyon, Lyon, France; 6 Institute of Pathology, East Site, Groupement Hospitalier Est, Hospices Civils de Lyon, Lyon, France

An accurate histological diagnosis of colorectal cancer is essential for promptly establishing appropriate therapeutic management. Furthermore, nowadays larger samples are needed to evaluate immunological and genetic panels. For malignant lesions that cannot be removed endoscopically, the European Guidelines recommend taking six carefully targeted biopsies from the suspected cancer focus 1 . Despite advancements in endoscopic imaging, a retrospective study of 962 patients undergoing colorectal adenocarcinoma resection found that 29% (62/217) required repeat endoscopy due to sampling errors 2 , leading to a 1.36-fold increase in time to surgery (95% CI 1.20–1.54, p<0.001) and higher healthcare costs. Choi et al. found that positive diagnosis rates for the first, second, and third biopsy specimens of advanced colorectal cancer were 78.1%, 87.5%, and 93.8%, respectively, with no significant increase from additional biopsies 3 . Reducing biopsy numbers by increasing targeted tissue sample size through cold snaring could enhance diagnostic sensitivity, minimize repeat procedures, and reduce pathologist workload.

We report a case of a 58-year-old patient who underwent a colonoscopy that revealed a 5-cm macronodular lesion proximal to the hepatic flexure ( Video 1 ). The lesion was thoroughly evaluated using white light imaging, narrow band imaging, and underwater magnification. The major 15-mm nodule was characterized by a pit pattern VN (Kudo classification), suggesting invasive cancer.

Procedure comparing cold snare biopsy with standard forceps biopsy in sampling colorectal cancer.Video 1

The lesion was considered non-removable endoscopically. Biopsies were performed, six with forceps and one with cold snaring. The six biopsies with forcepss were stored in formalin in one container, while the snare biopsy was placed in a separate container. Pathologists conducted a blind analysis.

Histological examination of the cold snare biopsy revealed focal areas with glandular fusion, papillary structures, and necrotic foci, affirming adenocarcinoma developed within a tubulovillous adenoma with high grade intra-epithelial neoplasia ( Fig. 1 ). In contrast, forceps biopsies showed only high grade intraepithelial neoplasia due to fragmentation and superficiality, without evidence of adenocarcinoma.

**Fig. 1:**
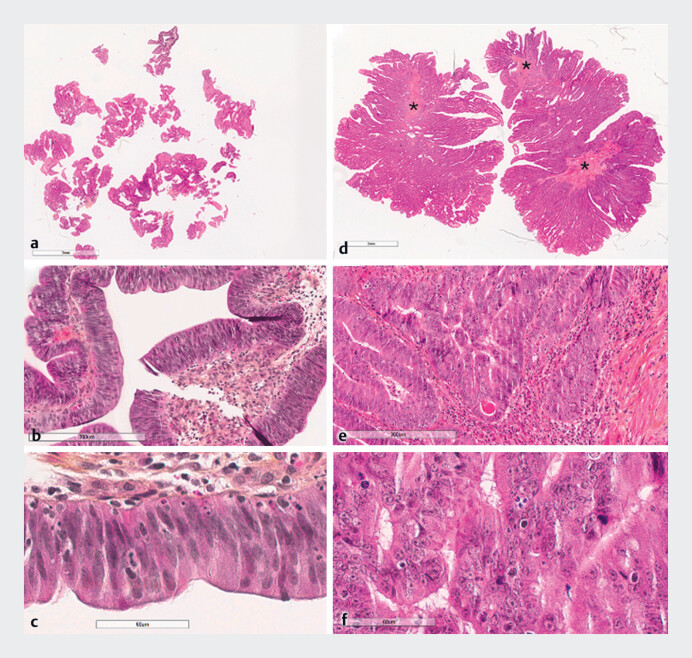
Comparison of histopathological features. **a–c** Forceps biopsy. **d–f** Cold snare biopsy.

Endoscopy_UCTN_Code_TTT_1AQ_2AC

## Notes

Endoscopy E-Videos https://eref.thieme.de/e-videos
*E-Videos* is an open access online section of the journal *Endoscopy* , reporting on interesting cases and new techniques in gastroenterological endoscopy. All papers include a high-quality video and are published with a Creative Commons CC-BY license. Endoscopy E-Videos qualify for HINARI discounts and waivers and eligibility is automatically checked during the submission process. We grant 100% waivers to articles whose corresponding authors are based in Group A countries and 50% waivers to those who are based in Group B countries as classified by Research4Life (see: https://www.research4life.org/access/eligibility/ ). This section has its own submission website at https://mc.manuscriptcentral.com/e-videos .

## References

[1] Pouw RE Bisschops R Endoscopic tissue sampling - Part 2: Lower gastrointestinal tract. European Society of Gastrointestinal Endoscopy (ESGE) Guideline Endoscopy 2021 53 1261 1273 10.1055/a-1671-6336 34715702

[2] Johnson GGRJ Hershorn O Sampling error in the diagnosis of colorectal cancer is associated with delay to surgery: a retrospective cohort study Surg Endosc 2022 36 4893 4902 34724583 10.1007/s00464-021-08841-z PMC8559691

[3] Choi Y Choi HS Optimal number of endoscopic biopsies in diagnosis of advanced gastric and colorectal cancer J Korean Med Sci 2012 27 36 39 10.3346/jkms.2012.27.1.36 22219611 PMC3247772

